# Ultrasound and Clinicopathological Features‐Based Machine Learning Model for Predicting Neoadjuvant Therapy Efficacy in Breast Cancer

**DOI:** 10.1002/cnr2.70600

**Published:** 2026-06-16

**Authors:** Tongtong Hao, Xiaohui Ji, Qianying Zhao, Mengying Wei

**Affiliations:** ^1^ Department of Ultrasound Medicine The Fourth Hospital of Hebei Medical University Shijiazhuang City Hebei Province China

**Keywords:** breast cancer, machine learning, neoadjuvant therapy, ultrasound

## Abstract

**Background:**

Accurate prediction of pathological complete response (pCR) after neoadjuvant therapy (NAT) remains challenging in breast cancer. Conventional imaging modalities, such as ultrasound and magnetic resonance imaging (MRI), have limited accuracy when used alone.

**Aims:**

To develop and validate a machine learning model integrating ultrasound imaging features and clinicopathological information for non‐invasive and individualized prediction of pCR following NAT in breast cancer patients.

**Methods and Results:**

This retrospective study included 609 breast cancer patients who underwent NAT. Ultrasound imaging features and clinicopathological variables were collected and analyzed. Data preprocessing was performed using Python and R. The diagnostic performance of ultrasound and MRI for predicting pCR was evaluated as a baseline. Significant predictors were identified through univariate and multivariate analyses. Three machine learning models—Random Forest, Logistic Regression, and Support Vector Machine—were developed and validated. Model performance was assessed using receiver operating characteristic (ROC) curves and decision curve analysis, while SHAP analysis and feature importance rankings were used to evaluate variable contributions. The Random Forest model achieved the best performance, with an AUC of 0.85 and an accuracy of 84.7%, outperforming conventional imaging assessments. Key predictors included early NAT tumor volume reduction ≥ 80%, increased echogenicity, HER2 positivity, and higher tumor‐infiltrating lymphocyte levels.

**Conclusion:**

The Random Forest model substantially improved prediction of pCR after NAT in breast cancer and may provide a practical, non‐invasive tool to support individualized treatment planning, and clinical decision‐making.

## 1 Introduction

Neoadjuvant therapy (NAT) for breast cancer is the first step of treatment for patients with breast cancer who do not have distant metastasis, using systemic therapy as the initial approach. The primary objectives of NAT include downstaging inoperable breast cancer to operable stages, converting non‐breast‐conserving cancer to breast‐conserving stages, and obtaining drug sensitivity information to guide subsequent treatment, thereby improving patient prognosis. Studies have shown that if pathological complete response (pCR) is achieved after neoadjuvant therapy, patients' disease‐free survival and overall survival will be prolonged. Therefore, pCR can serve as an alternative early clinical endpoint for long‐term survival in breast cancer patients [1]. However, among patients receiving NAT, fewer than 30% achieve pCR, and approximately 5% experience disease progression [2, 3]. Therefore, accurately assessing the likelihood of achieving pCR in the early phases of NAT is of critical clinical significance for improving surgical risk stratification and making timely treatment adjustments.

Currently, there is no standardized imaging criterion for predicting pathological complete response (pCR) following neoadjuvant therapy (NAT) in breast cancer patients [4]. Magnetic resonance imaging (MRI) offers high spatial resolution and is widely used in evaluating tumor response. Recent studies have explored radiomics and machine learning (ML)‐based models using multi‐region MRI features—including intratumoral, peritumoral, and background parenchymal enhancement (BPE)—to improve pCR prediction, achieving area under the curve (AUC) values over 0.85 in some cohorts [5, 6]. Similarly, models based on ^18F‐fluorodeoxyglucose positron emission tomography/computed tomography (^18F‐FDG PET/CT) have identified predictive radiomic features, although their clinical adoption is often limited by high cost and radiation exposure [7]. More recently, deep learning and radiomic models based on pretreatment breast ultrasound have shown promising performance, offering the advantages of accessibility and safety [8, 9]. Dual‐mode and temporal ultrasound fusion models have also emerged, capturing multi‐phase image changes to better reflect therapeutic response dynamics [10]. Despite these advances, most models still focus on baseline (pre‐NAT) imaging features and tend to operate as “black boxes,” often lacking transparency and interpretability, which hinders integration into daily clinical workflows.

Ultrasound remains a non‐invasive, real‐time, and cost‐effective imaging tool, but its standalone diagnostic performance in predicting pCR is suboptimal and highly operator‐dependent. Moreover, many current prediction models overlook dynamic imaging features that occur during treatment, such as tumor volume shrinkage and echogenicity changes, which may serve as early indicators of response. Understanding the full treatment trajectory rather than relying solely on static imaging provides a more comprehensive view of therapy efficacy [11, 12]. Furthermore, there is increasing demand for prediction models that balance accuracy with interpretability, as this is essential to gain clinicians' trust and facilitate clinical decision‐making.

Therefore, this study aims to develop a more accurate and robust predictive model for evaluating treatment response to neoadjuvant therapy in breast cancer patients, by integrating ultrasound imaging features and clinicopathological data using machine learning algorithms. Unlike previous studies that often depend solely on baseline features or adopt black‐box AI methods, our model incorporates dynamic early‐phase treatment information—such as tumor volume reduction and echogenicity variation—while employing interpretable tools such as SHAP values to increase model transparency. By doing so, we provide a non‐invasive, explainable, and clinically practical tool that can aid early prediction of pCR, guide individualized treatment adjustments, and support real‐time clinical decision‐making.

## 2 Methods

### 2.1 Study Subjects

This study retrospectively collected data on breast cancer patients who underwent neoadjuvant therapy (NAT) at the Fourth Hospital of Hebei Medical University from January 2019 to February 2024. Inclusion criteria included: (1) treatment plans based on breast cancer clinical guidelines, encompassing chemotherapy, targeted therapy, and immunotherapy; (2) surgery performed post‐NAT with postoperative pathological results obtained; (3) all patients underwent ultrasound examination and histological biopsy prior to NAT; (4) for patients with multiple lesions, the largest tumor was analyzed. Exclusion criteria were: (1) poor image quality; (2) patients who received radiotherapy, endocrine therapy, or other treatments prior to NAT; (3) baseline ultrasound examinations prior to NAT where the tumor was not clearly visible, such as in cases of inflammatory breast cancer, large breast cancer, and breast cancer with skin ulceration. A total of 609 patients were included in the final analysis. Among all eligible patients during the study period, all eligible pCR patients were included, while 119 non‐pCR patients were randomly selected from the available non‐pCR population for comparative analysis. This sampling strategy was adopted to provide sufficient representation of the pCR class during model development, although the resulting dataset does not reflect the true clinical prevalence of pCR. To reduce the impact of class imbalance, class weights inversely proportional to class frequencies were applied during the training of the Random Forest, Logistic Regression, and Support Vector Machine models.

**FIGURE 1:**
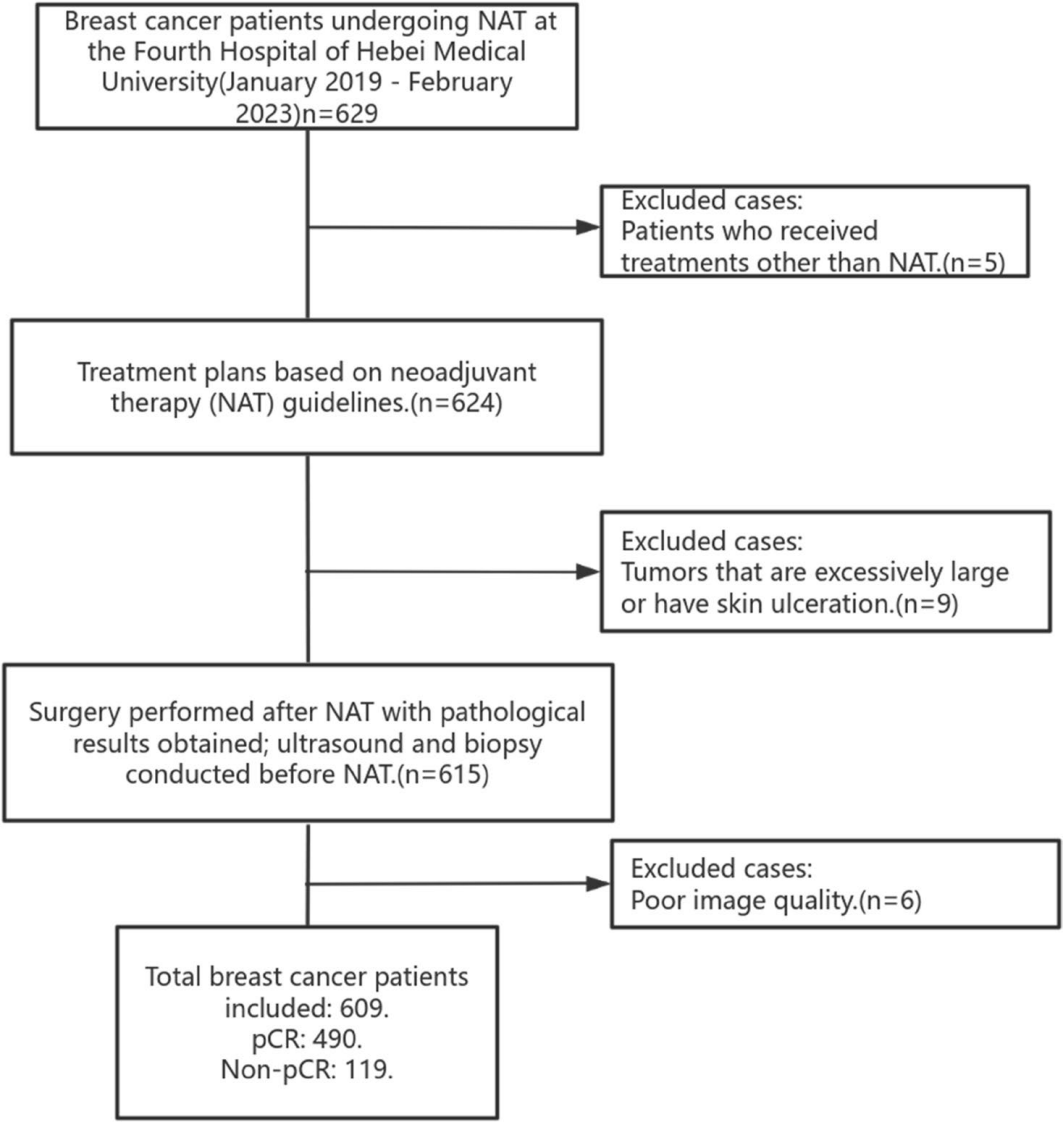
Inclusion and exclusion criteria for study subjects.

The detailed inclusion and exclusion process is illustrated in Figure 1. All included patients met the eligibility criteria and had complete clinical and pathological data available. This retrospective study was approved by the Ethics Committee of the Fourth Hospital of Hebei Medical University (Approval No. 2024KS207), and the requirement for informed consent was waived.

### 2.2 Evaluation Criteria

Patient clinical and pathological data were collected, including age, estrogen receptor (ER), progesterone receptor (PR), human epidermal growth factor receptor‐2 (HER‐2), Ki‐67 proliferation index, p53, and levels of tumor‐infiltrating lymphocytes (TILs).

According to the St. Gallen International Expert Consensus, tumors are classified into four molecular subtypes: Luminal A (ER+, PR ≥ 20%, HER2‐, Ki‐67 < 20%), Luminal B (ER+, PR < 20% or HER2+ or Ki‐67 ≥ 20%), HER2‐positive (HER2+ and ER−, PR−), and triple‐negative (ER−/HER2− and PR−). ER positivity is defined as nuclear staining in ≥ 1% of tumor cells, and high Ki‐67 expression is defined as ≥ 20% [13]. The assessment area for TILs includes the borders of invasive tumors and immune infiltration in adjacent normal tissues, with all stromal mononuclear cells not in direct contact with cancer cells categorized as TILs. TILs are classified into three levels: low (0%–9%), moderate (10%–49%), and high (≥ 50%) [14].

The staging of breast cancer follows the 2017 AJCC 8th edition criteria: Stage 0 is carcinoma in situ; Stage I is an early‐stage tumor (T1N0M0); Stage II is characterized by a slightly larger tumor or involvement of axillary lymph nodes without distant metastasis; Stage III is locally advanced, with significantly enlarged tumors or multiple lymph node involvement, possibly spreading to the chest wall or skin; Stage IV is advanced, with distant metastasis (M1) [15].

According to the Miller & Payne grading system [16], pathological response to neoadjuvant chemotherapy (NAC) was assessed, with Grade 5 defined as the pCR group and Grades 1–4 as the non‐pCR group. In this study, pCR was defined as the absence of invasive cancer in the breast, with or without residual ductal carcinoma in situ, and negative regional lymph nodes (ypT0/is ypN0). Patients in the pCR group met this definition. This study focused on factors associated with pCR in the primary breast tumor.

#### 2.2.1 Equipment and Image Selection Criteria

Ultrasound examinations in this study were conducted using PHILIPS EPIQ Elite, SIEMENS ACUSON Sequoia Silver, and SIEMENS ACUSON 3000 with high‐frequency linear array probes, with a frequency range of 7.5 to 15 MHz. Ultrasound data collection included tumor characteristics prior to neoadjuvant therapy (NAT), covering maximum diameter (≥ 3 cm or < 3 cm), shape (regular or irregular), edge (clear or unclear), and changes in echogenicity within the tumor before and after NAT (increased, decreased, no change, or disappeared). The echogenic characteristics of the tumor are referenced against breast fat: if the tumor's echogenicity is higher than that of breast fat, it is considered hyperechoic; if it is lower than that of subcutaneous fat, it is considered hypoechoic.

Based on the NAT treatment cycles, treatment is divided into early (1–2 cycles), mid (2–4 cycles), and late stages (over 4 cycles). At each stage, we calculated the maximum diameter reduction rate (≥ 30% or < 30%) and the volume reduction rate (≥ 80% or < 80%). The formula for volume calculation is V=π/4×LWH, where *L* is the length, *W* is the width, and *H* is the height. The reduction rate in volume or diameter between two cycles is calculated using the formula *X* = [V(*n*−2)−*V*(*n*)]/*V*(*n*−2), where n represents the treatment cycle. According to the Response Evaluation Criteria in Solid Tumors (RECIST), the complete disappearance of a tumor is defined as a clinical complete response (cCR) [17]. In this study, MRI complete response (mCR) was defined as the absence of abnormal enhancement in the original tumor region on dynamic contrast‐enhanced T1‐weighted imaging (T1WI). For ultrasound, the absence of a detectable lesion was considered an ultrasound complete response (uCR).

All breast ultrasound and MRI images were independently reviewed by two radiologists, each with more than 5 years of experience in breast imaging, and blinded to the pathological outcomes. In cases of disagreement, a third, more senior radiologist made the final decision.

### 2.3 Statistical Analysis

Statistical analyses were performed using Python (version 3.7) and R (version 4.4.1). Quantitative data were analyzed using the Mann–Whitney *U* test, while categorical data were analyzed using the chi‐squared test. Significant clinical, pathological, and ultrasonic features associated with pCR were identified through univariate and multivariate analyses. A *p* value of less than 0.05 was considered statistically significant. In this study, the missing rates were approximately 19% for Tumor‐Infiltrating Lymphocytes (TILs), 17% for p53, and 15% for the reduction rates of mid‐stage and late‐stage tumors after neoadjuvant therapy (NAT).

Due to the potential clinical significance of these variables, they were retained in the analysis, and missing values were handled using multiple imputation methods based on clinically relevant predictors and outcome‐related information. For the remaining variables, which had less than 5% missing data, mode imputation was applied to categorical variables and mean imputation to continuous variables because the expected impact on bias was considered minimal. Sensitivity analysis suggested that model performance remained generally consistent before and after imputation, indicating that missing‐data handling did not substantially influence the overall results.

## 3 Model Development and Evaluation

### 3.1 Model Development

Three machine learning algorithms were employed to predict pathological complete response (pCR) status: Logistic Regression, Random Forest, and Support Vector Machine (SVM). Based on univariate and multivariate analyses, significant ultrasound and clinicopathological features associated with pCR were identified and used as input variables. The dataset was divided into a training set (*n* = 426) and a validation set (*n* = 183) using a stratified 7:3 ratio to preserve the relative proportions of pCR and non‐pCR cases. To mitigate the impact of class imbalance, class weights were automatically assigned inversely proportional to class frequencies using the “balanced” option in scikit‐learn during the training of the Random Forest, Logistic Regression, and Support Vector Machine models. To prevent data leakage and ensure the integrity of model evaluation, feature selection was performed exclusively on the training set. Model development was conducted using the training set. Detailed modeling procedures, including parameter tuning strategies and key model coefficients, are provided in Table S1.

### 3.2 Model Performance Evaluation

Each model (Random Forest, Logistic Regression, and SVM) trained on the training set was validated using the validation set, applying the same predictor variables and preprocessing steps as in the training phase. The optimal probability threshold for predicting pCR was determined by maximizing Youden's J statistic based on the ROC curve for each model. Detailed procedures and threshold values are provided in the Supporting Information. These predictions were then used to compute receiver operating characteristic (ROC) curves, area under the curve (AUC), sensitivity, specificity, and accuracy.

### 3.3 Feature Importance Analysis

To quantify the relative importance of significant features in model decision‐making, we employed two methods: (1) Random Forest feature importance ranking, which evaluates the contribution of features to predictions by calculating the total reduction in impurity during node splits; and (2) SHAP value analysis, which measures the marginal contribution of features to the model output using SHAP values and visualizes and interprets the key features of the optimal model through SHAP waterfall plots.

### 3.4 Decision Curve Analysis

Decision Curve Analysis (DCA) was used to evaluate the clinical utility of different predictive models. DCA calculates the net benefit of a model at various risk thresholds, providing insight into whether a specific model can improve clinical decision‐making and lead to greater clinical benefits.

## 4 Results

### 4.1 Diagnostic Performance of Ultrasound and MRI

**FIGURE 2:**
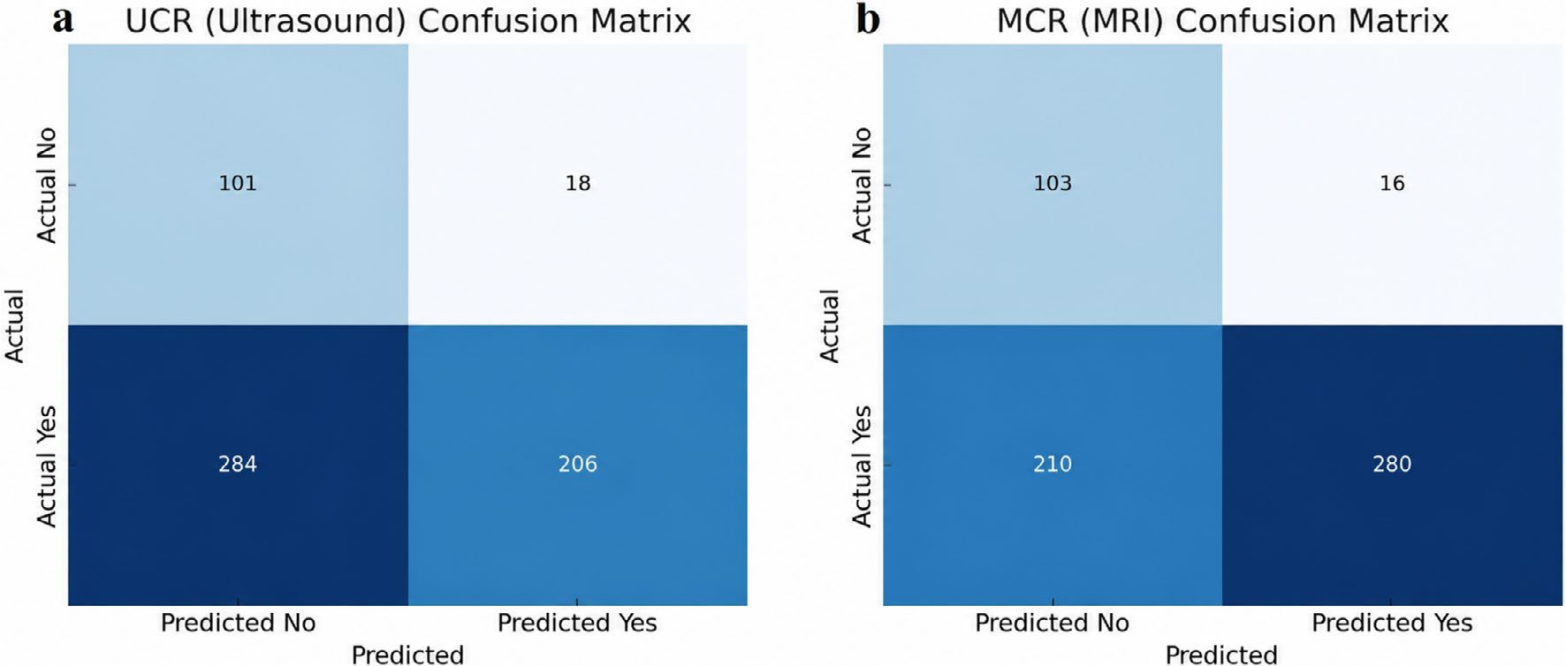
Confusion matrix for ultrasound and MRI. (a) Confusion matrix for ultrasound and (b) confusion matrix for MRI.

**FIGURE 3:**
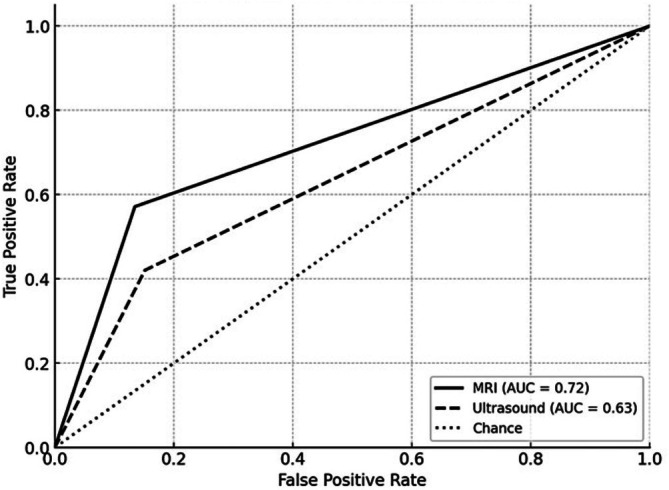
ROC curve for MRI and ultrasound.

The confusion matrix showed that ultrasound had an overall accuracy of 50.4% (307/609), a sensitivity of 42.0% (206/490), a specificity of 84.9% (101/119), and an area under the curve (AUC) of 0.64. For MRI, the diagnostic performance included an accuracy of 62.9% (383/609), sensitivity of 57.1% (280/490), specificity of 86.6% (103/119), and an AUC of 0.72. Refer to the corresponding confusion matrices in Figure 2a,b, and to the ROC curve comparison in Figure 3.

### 4.2 Patient Characteristics

The univariate analysis (Tables 1 and 2) revealed statistically significant differences in tumor volume and maximum diameter reduction rates during NAT, residual tumor size, changes in echogenicity before and after NAT, and tumor shape on ultrasound (all *p* < 0.05). Multivariate analysis identified the following factors as independently associated with pCR: early‐NAT tumor volume reduction ≥ 80% (OR = 30.80, *p* < 0.001), increased echogenicity after NAT (OR = 5.16, *p* < 0.001), tumor disappearance (OR = 17.37, *p* = 0.001), triple‐negative breast cancer (OR = 4.29, *p* < 0.001), HER‐2 positive breast cancer (OR = 5.93, *p* < 0.001), and intermediate/high levels of TILs (OR = 2.97, *p* < 0.001).

**TABLE 1:** Univariate and multivariate analysis (ultrasound features).

Characteristics	pCR^a^ (*N* = 490)	Non‐pCR (*N* = 119)	Univariate analysis		Multivariate analysis		
			χ^2^	*p*	OR^b^	Waldχ^2^	*p*
PreNAT^c^ Tumor maximum diameter^d^			0.237	0.626			
> 3 cm	270(55.1%)	62(52.1%)					
≤ 3 cm	220(44.9%)	57(47.9%)					
Residual Tumor maximum diameter^d^			26.147	< 0.001		0.552	0.457
> 1 cm	321(65.5%)	107(89.9%)			*Ref*		
≤ 1 cm	169(34.5%)	12(10.1%)			1.56		
Early‐NAT Reduction Rate of minimum diameter^d^			0.272	0.601			
≤ 30%	106(21.6%)	29(24.4%)					
> 30%	384(78.4%)	90(75.6%)					
Reduction Rate of maximum diameter^d^							
Early‐NAT			10.776	< 0.001		2.679	0.102
< 30%	220(44.9%)	74(62.2%)			*Ref*		
≥ 30%	270(55.1%)	45(37.8%)			0.61		
Mid‐NAT			6.986	0.008		0.827	0.363
< 30%	323(65.9%)	94(79.0%)			*Ref*		
≥ 30%	167(34.1%)	25(21.0%)			0.74		
Late‐NAT			9.983	0.002		0.544	0.461
< 30%	343(70.0%)	101(84.9%)			*Ref*		
≥ 30%	147(30.0%)	18(15.1%)			0.71		
Reduction Rate of Volume^d^							
Early‐NAT^e^				< 0.001		29.485	< 0.001***
< 80	265(54.1%)	116(97.5%)			*Ref*		
≥ 80%	225(45.9%)	3(2.5%)			30.81		
Mid‐NAT			10.283	0.001		0.459	0.497
< 80	383(78.2%)	109(91.6%)			*Ref*		
≥ 80%	107(21.8%)	10(8.4%)			1.41		
Late‐NAT			15.936	< 0.001		0.245	0.876
< 80	374(76.3%)	111(93.3%)			*Ref*		
≥ 80%	116(23.7%)	8(6.7%)			0.84		
Edge			3.290	0.069			
Clear	212(43.3%)	40(33.6%)					
Unclear	278(56.7%)	79(66.4%)					
Shape			9.925	0.002			
Irregular	244(49.8%)	79(66.4%)					
Regular	246(50.2%)	40(33.6%)					
Echo^e^				< 0.001		26.362	< 0.001***
No change	249(50.8%)	100(84.0%)			*Ref*		
Disappearance	93(19.0%)	1(0.8%)			17.37		0.001***
Higher	139(28.4%)	12(10.1%)			5.16		< 0.001***
Lower	9(1.8%)	6(5.0%)			0.44		0.194

*Note:* All variables were analyzed after imputation of missing values. Numerical data are presented as medians, with interquartile ranges in parentheses. Count data are presented as the number of lesions (percentage). Variables with *p* < 0.05 in univariate analyses were included in multivariate analyses. Odds ratios (OR) and *p* values are determined by multivariate logistic regression.

^a^ pCR: Pathological complete response.

^b^ OR: Odds ratio.

^c^ NAT: Neoadjuvant therapy.

^d^ The cutoff values are determined by the maximum Youden index.

^e^ Fisher's exact test is used.

^***^ *p* < 0.001.

**TABLE 2:** Univariate and multivariate analysis (clinicopathological features).

Characteristics	pCR^a^ (*N* = 490)	Non‐pCR (*N* = 119)	Univariate analysis		Multivariate analysis		
			χ^2^	*p*	OR^b^	Waldχ^2^	*p*
Age, Mean(SD), years	49.99(10.86)	51.27(9.75)		0.22			
p53, Mean(SD)	37.10(32.39)	28.85(31.23)		0.01	1	0.574	0.449
Ki67			10.171	0.001		0.448	0.503
< 20%	76(15.5%)	34(28.6%)			*Ref*		
≥ 20%	414(84.5%)	85(71.4%)			1.37		
stage			2.337	0.126			
≥ II	192(39.2%)	37(31.1%)					
II	298(60.8%)	82(68.9%)					
subtype			61.827	< 0.001		26.691	< 0.001***
Luminal	61(12.4%)	51(42.9%)			*Ref*		
HER‐2	312(63.7%)	57(47.9%)			4.29		< 0.001***
Triple negative	117(23.9%)	11(9.2%)			5.93		< 0.001***
TILs^c^			54.462	< 0.001		16.822	< 0.001***
Low	148(30.2%)	80(67.2%)			*Ref*		
Intermediate/high	342(69.8%)	39(32.8%)			2.97		

*Note:* All variables were analyzed after imputation of missing values. Numerical data are presented as medians, with interquartile ranges in parentheses. Count data are presented as the number of lesions (percentage). Variables with *p* < 0.05 in univariate analyses were included in multivariate analyses. Odds ratios (OR) and *p* values are determined by multivariate logistic regression.

^a^ pCR: Pathological complete response.

^b^ OR: Odds ratio.

^c^ TILs: Tumor infiltrating lymphocytes.

^***^ *p* < 0.001.

### 4.3 Comparison of Three Predictive Model Performances

No statistically significant differences were observed in the distributions of key clinical, pathological, and imaging variables between the training and validation cohorts (all *p* > 0.05; Cramer's *V* < 0.1), indicating good baseline comparability. Additionally, the pCR rates were consistent between the two sets. Full statistical results are provided in Table S2.

**FIGURE 4:**
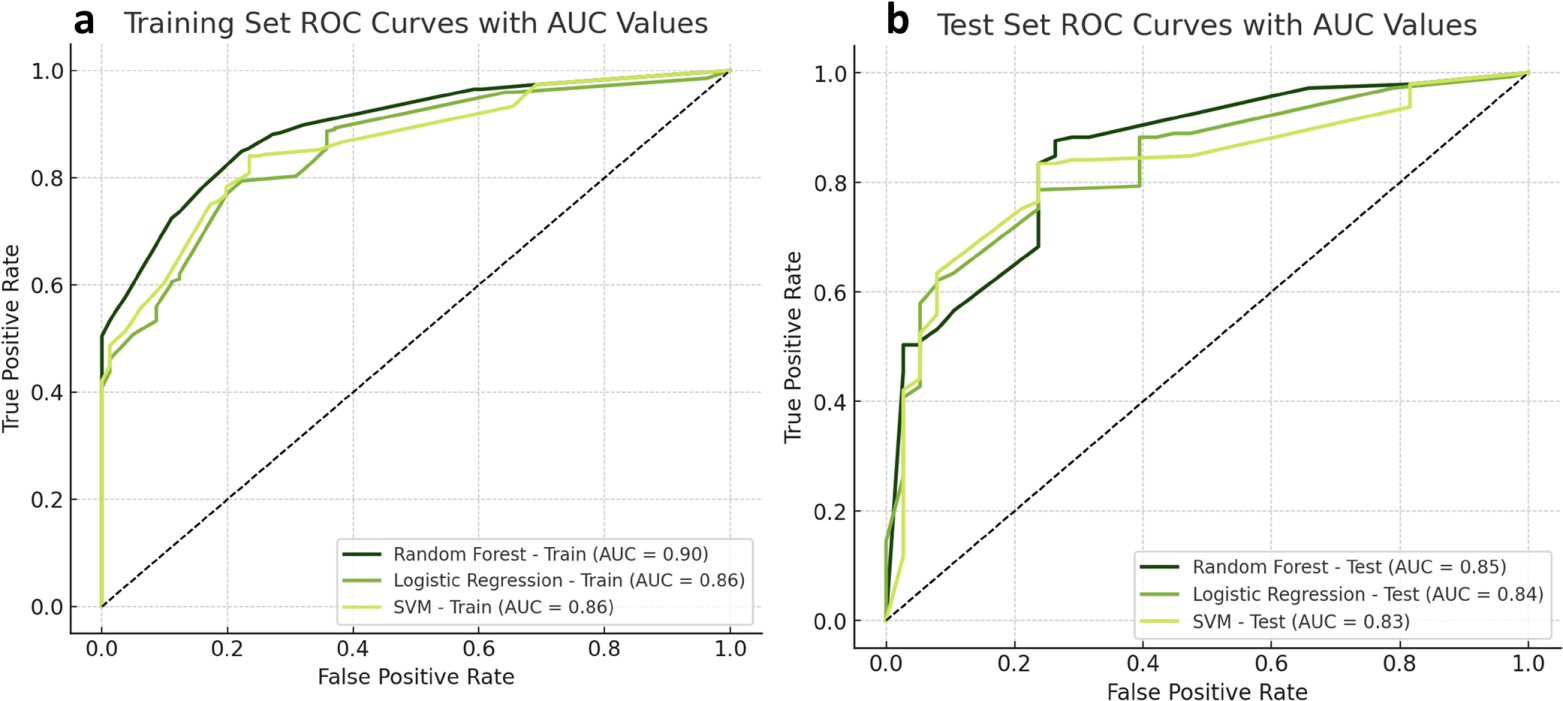
Comparison of ROC curves for training and validation sets. (a) Comparison of ROC curves for training sets and (b) comparison of ROC curves for validation sets.

**FIGURE 5:**
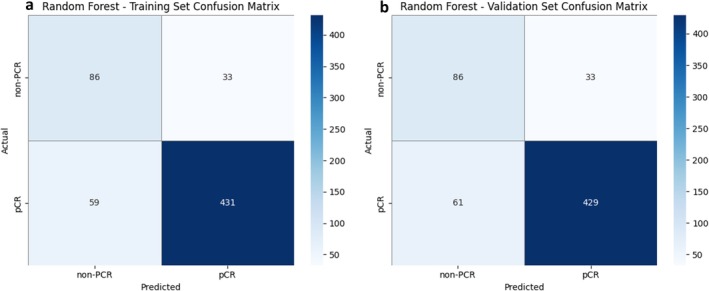
Confusion matrix for the random forest model. (a) The training set and (b) the validation set.

Comparisons of the Random Forest, SVM, and Logistic Regression models (Figure 4a,b, Table 3) revealed that the Random Forest model outperformed the others in terms of AUC, sensitivity, and accuracy. Specifically, the Random Forest model achieved an AUC of 0.90 in the training set and 0.85 in the validation set, with a sensitivity of 87.6% and an accuracy of 84.7% in the validation set, all of which were superior to those of the SVM and Logistic Regression models. Although the Random Forest model showed slightly lower specificity compared to the other models, it demonstrated the strongest overall predictive performance and stability. The confusion matrix of the Random Forest model is shown in Figure 5.

**TABLE 3:** Comparison of model performance.

Model	Training cohort					Validation cohort				
	AUC^a^	95% CI^b^	Accuracy	Sensitivity	Specificity	AUC	95% CI	Accuracy	Sensitivity	Specificity
Random Forest	0.90	0.792~0.864	85.2%	88.1%	72.8%	0.85	0.788~0.894	84.7%	87.6%	72.8%
Logistic Regression	0.86	0.752~0.829	79.1%	79.4%	77.7%	0.84	0.721~0.841	78.1%	78.6%	76.3%
SVM^c^	0.86	0.818~0.885	82.2%	83.5%	76.5%	0.83	0.801~0.903	82.0%	83.4%	76.3%

^a^ AUC: Area under the receiver operating characteristic curve.

^**b**^ CI: Confidence intervals.

^c^ SVM: Support vector machine.

### 4.4 Feature Importance Analysis

**FIGURE 6:**
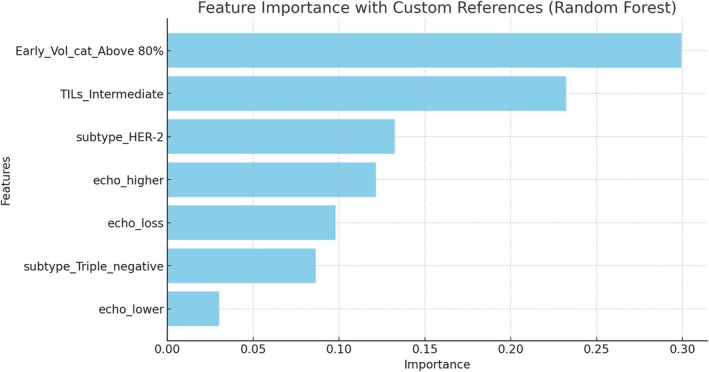
Random forest feature importance ranking. This figure displays the importance scores of each feature for model prediction outcomes. Importance scores are typically measured based on the degree to which a feature improves model accuracy. The most critical feature is early‐NAT tumor volume reduction ≥ 80%, followed by intermediate/high levels of TILs expression and HER‐2 positive breast cancer.

**FIGURE 7:**
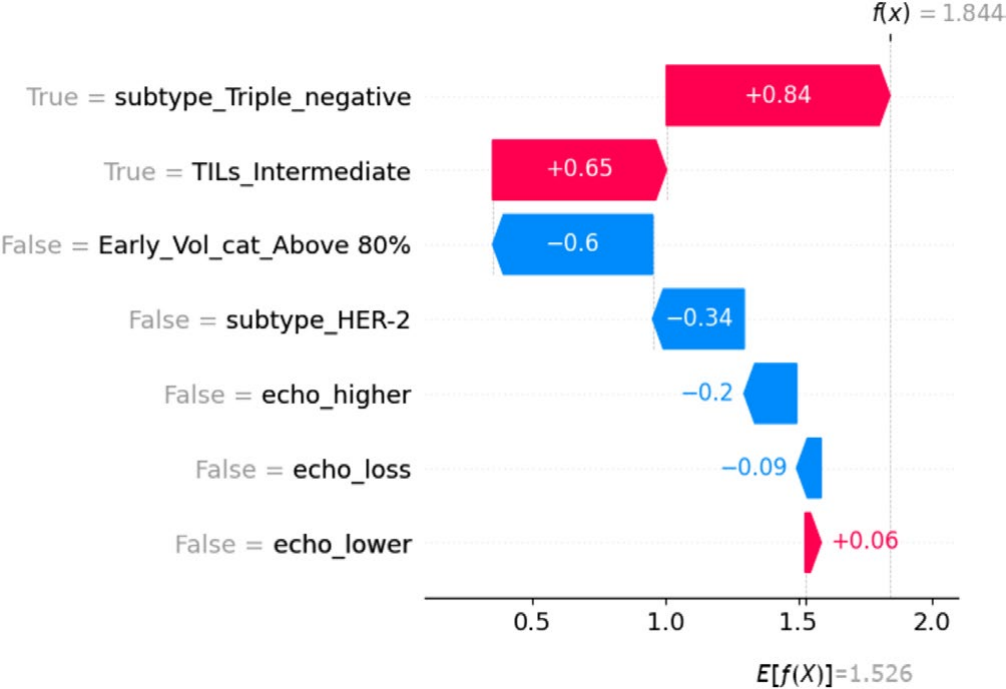
SHAP waterfall plot. Red blocks represent positive impacts of features on the prediction outcome, while blue blocks represent negative impacts. In the waterfall plot, the cumulative contributions of each feature based on the average predicted value *Ef*(*x*) are summed to obtain the predicted value for the sample (*f*(*x*) value displayed on the right). For instance, in Case 1, the model predicts an 85.7% probability of pCR for the patient, consistent with the pathological results. ✱The formula for calculating the prediction probability is: $\text{Probability}=\frac{1}{1+e^{-f\left(x\right)}}$.

**FIGURE 8:**
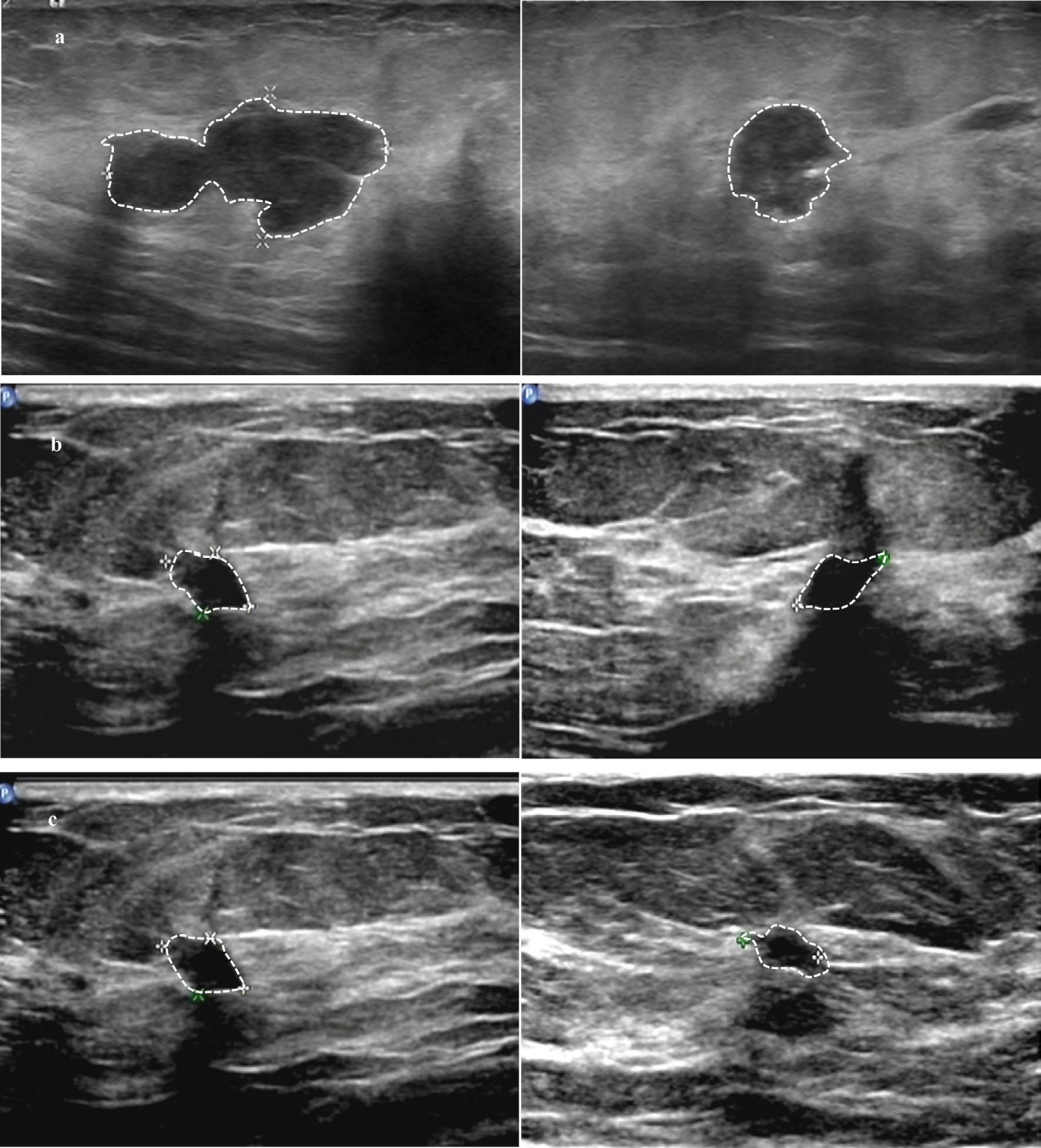
Case 1. A 64‐year‐old female with a left breast mass, classified as BI‐RADS category 5, biopsy confirmed as invasive ductal carcinoma, HER‐2 positive breast cancer (ER 90%, PR 40%, HER‐2 3+), and TILs at 30%. She received six cycles of neoadjuvant therapy. Ultrasound imaging before NAT (a) showed the tumor as hypoechoic. No significant changes in echogenicity were observed during the 2nd (b) and 6th (c) cycles of NAT, with a tumor volume reduction rate of 77% after the second cycle. Pathology after breast‐conserving surgery confirmed a pathological complete response (pCR).

Figure 6 displays the importance scores of significant features for model prediction outcomes. Importance scores are typically measured based on the degree to which a feature improves model accuracy. The most critical feature is early‐NAT tumor volume reduction ≥ 80%, followed by intermediate/high levels of TILs expression and HER‐2 positive breast cancer. SHAP values explain model outputs by quantifying the marginal contribution of each feature to the prediction for individual samples. For a single patient, these results illustrate the impact of different features on the model's prediction of clinical outcomes (Figures 7 and 8).

### 4.5 Decision Curve Analysis

**FIGURE 9:**
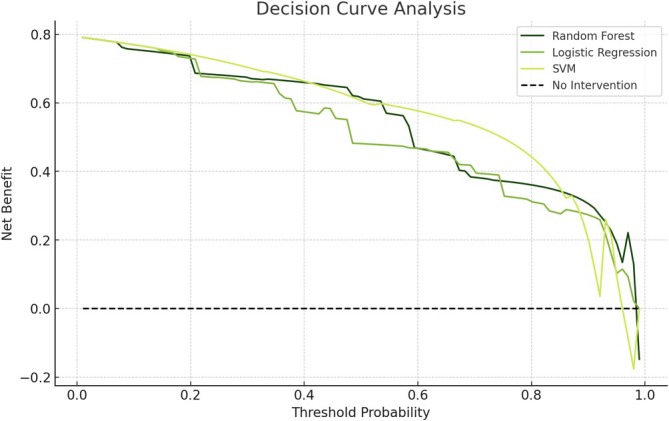
Decision curve analysis the random forest model demonstrates a higher net benefit under a broader range of high‐risk thresholds.

**FIGURE 10:**
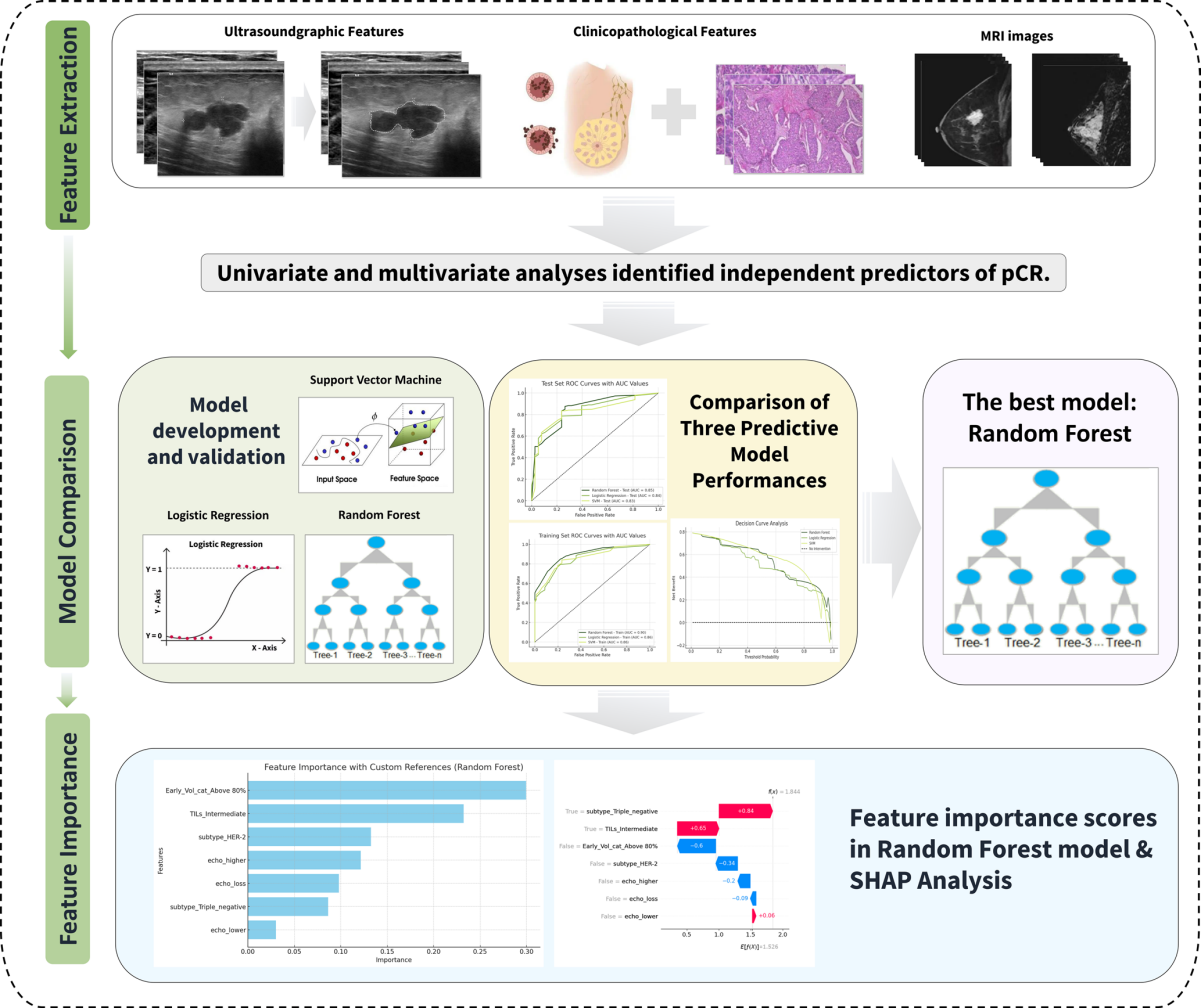
Feature selection and model construction flowchart.

The decision curve analysis results for the three models indicate that the Random Forest model is more beneficial for predicting pCR in breast cancer patients post‐NAT within a higher risk threshold range (Figure 9). The flowchart of feature selection and model construction is shown in Figure 10.

## 5 Discussion

### 5.1 The Potential of Machine Learning in Predicting pCR

Ultrasound and magnetic resonance imaging (MRI) are the primary imaging modalities for evaluating the efficacy of neoadjuvant therapy (NAT) in breast cancer and have been widely implemented in clinical practice. Previous studies have reported a sensitivity of 60.8% and specificity of 78.0% for ultrasound in predicting pathological complete response (pCR) [3], whereas in our study, the sensitivity and specificity were 42.0% and 84.9%, respectively. The diagnostic performance of ultrasound is influenced by multiple factors, particularly post‐chemotherapy tissue changes, such as the reduction of cancer cells accompanied by scar formation, which makes it difficult for ultrasound to distinguish residual tumor from fibrotic tissue. Moreover, ultrasound findings are highly operator‐dependent, further compromising reproducibility and consistency. MRI offers certain advantages in soft tissue imaging; however, its reliability in predicting pCR remains controversial. Literature has reported MRI sensitivity and specificity of 75% and 67%, respectively, with a positive predictive value of only 48% [18]. In our study, MRI showed a sensitivity of 57.1% and a specificity of 86.6%. Stromal neovascularization in the tumor region following NAT may lead to exaggerated contrast enhancement, resulting in false positives; conversely, signal attenuation due to anti‐angiogenic therapy can cause false negatives. Furthermore, MRI relies on gadolinium‐based contrast agents, and repeated use may lead to gadolinium deposition in the nervous system [19], posing a potential safety concern for patients. Although both ultrasound and MRI have their respective strengths, relying solely on either modality is insufficient for accurately predicting pCR.

Recent advances in radiomics and artificial intelligence have explored more sophisticated imaging‐based methods for predicting treatment response. For instance, models built on multi‐region MRI radiomics—including tumor, peritumoral, and background parenchymal regions—have achieved AUCs up to 0.861 [5], and when combined with clinical data, deep learning frameworks have shown improved prediction of early response, especially in subtypes such as triple‐negative breast cancer (5). Similarly, ultrasound‐based deep learning radiomics models have demonstrated excellent performance, with reported AUCs as high as 0.96 in multicenter validations [8], and bimodal ultrasound models have further leveraged grayscale and Doppler signals to capture dynamic tumor characteristics [9]. Furthermore, PET/CT radiomic features, though less accessible, have also been shown to be predictive of pCR, particularly when integrated with clinical variables [7]. These studies highlight the potential of various imaging modalities and advanced ML‐based models; however, many rely heavily on either pretreatment imaging or high‐cost modalities, limiting their routine clinical application. Notably, several recent works have emphasized the value of dynamic or longitudinal imaging features—such as early‐treatment changes in size, contrast, or texture—for improving prediction accuracy [6, 10]. This supports the strategy adopted in our study: incorporating early changes in tumor volume and echogenicity during NAT via ultrasound to reflect treatment response in real time.

In this study, three machine learning models—random forest, logistic regression, and support vector machine (SVM)—were compared. The Random Forest model achieved the highest performance, with an AUC of 0.90 in the training set and 0.85 in the validation set. Its advantages include the ability to capture complex interactions among variables, strong robustness, and suitability for high‐dimensional, heterogeneous data, while being relatively less prone to overfitting. Logistic Regression is simpler and more interpretable, but it cannot fully capture the complex variable interactions present in our dataset, which leads to lower predictive accuracy. Compared with more complex radiomics approaches, the models used in this study extracted key features directly from routinely obtainable ultrasound images, reducing data acquisition costs and improving clinical practicality.

The Random Forest model provided strong clinical net benefit in decision curve analysis and reliably stratifies patients into higher‐ and lower‐risk groups. However, the raw predicted probabilities from the Random Forest should be interpreted with caution when applied to individual patients. This is because Random Forest probabilities are derived from the proportion of trees voting for a class, which may not precisely correspond to the true likelihood of achieving pCR in a single patient. Consequently, while these probabilities are suitable for population‐level risk stratification and decision‐support, they are currently not recommended for exact individualized probability counseling. Future work will explore post hoc calibration methods, such as Platt scaling or isotonic regression, to improve agreement between predicted and observed probabilities while preserving the model's discriminative performance.

Importantly, the present study differs from many existing imaging‐based prediction models in several aspects. First, rather than relying solely on pretreatment static imaging features, our model incorporated dynamic early‐treatment ultrasound changes, including tumor volume reduction and echogenicity variation during NAT, which may better reflect real‐time tumor sensitivity. Second, unlike many deep learning or radiomics “black‐box” systems, our model emphasized interpretability through SHAP analysis and feature importance ranking. SHAP waterfall plots visualize how each feature contributes to individual predictions, highlighting key variables and providing personalized decision support. Third, by using routinely obtainable ultrasound and clinicopathological features, the model may offer greater practicality, accessibility, and cost‐effectiveness compared with more resource‐intensive imaging modalities such as MRI or PET/CT.

### 5.2 Significant Variable Analysis

This study included a relatively large sample size compared with several previous studies, enhancing the reliability and representativeness of the results. Our findings are generally consistent with existing literature, which not only validates the effectiveness of the research methods and results but also enhances the generalizability and reproducibility of these findings. For each significant variable, we systematically explore its potential mechanisms and clinical significance.

#### 5.2.1 Tumor‐Infiltrating Lymphocytes (TILs)

Evidence has shown that intermediate/high levels of TILs expression are closely associated with the probability of pCR, particularly in triple‐negative breast cancer (TNBC) and HER‐2 positive breast cancer, where higher TILs levels are significantly correlated with higher pCR rates [20, 21]. The CTNeoBC meta‐analysis further confirmed that, in TNBC and HER‐2 positive patients with higher TILs levels before NAT, the pCR rate could increase by approximately four times [2]. The increase in tumor‐infiltrating lymphocytes (TILs) can be explained by neoadjuvant chemotherapy (NAC) inducing the release of damage‐associated molecular patterns (DAMPs) through the destruction of cancer cells [22]. Compared to other biomarkers, only TILs were independently associated with pCR in this study, making TILs a relatively robust biomarker.

#### 5.2.2 Tumor Volume Reduction Rate

Assessment of tumor size remains a widely accepted and intuitive approach for evaluating the efficacy of neoadjuvant therapy (NAT). While previous studies have suggested that reductions in short‐axis diameter may better reflect treatment response than long‐axis changes [23, 24], our study found that the early‐NAT tumor volume reduction ≥ 80% was significantly associated with pCR, showing superior predictive value compared to unidimensional measurements. Specifically, the Early‐NAT Reduction Rate of minimum diameter, measured after the second cycle of NAT, was not significantly associated with pCR, suggesting that early changes in minimum diameter may have limited predictive value at this stage. In contrast, volumetric changes demonstrated stronger predictive performance. Consistent with findings by Dobruch‐Sobczak et al., who reported significant tumor volume reduction after the first and fourth cycles of NAT [25], our analysis across the full treatment course showed that the Reduction Rate of Volume, particularly after the second cycle, was significantly associated with pCR. It outperformed both the reduction rate of maximum diameter and the early‐NAT reduction rate of minimum diameter in predicting therapeutic response. Notably, tumors often undergo morphological alterations during NAT, such as becoming flatter or more irregular, which can compromise the accuracy of unidimensional measurements. In contrast, volumetric assessments more comprehensively reflect three‐dimensional tumor changes. For patients who do not exhibit significant early‐NAT tumor volume reduction, clinicians may need to consider adjusting the therapeutic regimen or incorporating alternative strategies.

#### 5.2.3 Increased Echogenicity

Echogenic changes are another simple and easily obtainable parameter in ultrasound images. Studies have shown that increased echogenicity can serve as an effective ultrasound feature for predicting treatment outcomes, while persistent hypoechogenicity often indicates a poor response to treatment [26]. This is consistent with the findings of this study. Histological studies have observed changes such as fibrosis at the tumor site in excised specimens after NAT [23], which are primarily related to the heterogeneity of tumor cells. After NAT, tumor tissue is replaced by scar and fibrous tissue due to fibrosis, fragmentation, and/or necrosis, leading to a reduction in the number of tumor cells. This transformation is reflected as increased echogenicity on ultrasound.

#### 5.2.4 Molecular Subtyping of Breast Cancer

Research has shown that the efficacy of neoadjuvant therapy (NAT) for breast cancer is associated with molecular subtypes. Triple‐negative breast cancer (TNBC) and HER‐2 positive breast cancer are more likely to achieve pathological complete response (pCR) during NAT and are associated with longer disease‐free survival compared to Luminal‐type breast cancer [27]. TNBC exhibits high proliferative activity and a higher density of tumor‐infiltrating lymphocytes (TILs), particularly CD8+ T cells, which enhance the efficacy of chemotherapy. The favorable response of HER‐2 positive breast cancer is attributed to HER‐2 targeted therapies (such as trastuzumab and pertuzumab), which inhibit tumor growth and improve therapeutic outcomes.

### 5.3 Limitations and Future Directions

This study has several limitations. First, it was conducted using data from a single center, which may restrict the generalizability of our findings. Future research should involve prospective multi‐center external validation using independent cohorts from at least 2–3 institutions with diverse patient populations, imaging equipment, and clinical settings. A larger validation sample of approximately 500–1000 patients would provide a more robust assessment of model performance. Key evaluation metrics should include discrimination (AUC, sensitivity, specificity), calibration, and clinical utility through decision curve analysis. Such validation will be essential to assess the robustness, calibration, and generalizability of the model before clinical implementation.

Second, the dataset included a higher proportion of pCR cases, as all eligible pCR patients during the study period were included, while only a randomly selected subset of non‐pCR cases was analyzed. Although class weighting was applied during model training to reduce the impact of class imbalance, this sampling strategy may still introduce selection bias, affect probability calibration, and limit generalizability to populations with different pCR prevalence.

Third, manual interpretation of ultrasound features by experienced physicians reflects current clinical workflows but may introduce subjectivity and inter‐observer variability. Although double review and senior adjudication were applied to improve consistency, differences in experience across institutions may still affect reproducibility and model generalizability. Future studies could adopt standardized protocols and automated or semi‐automated feature extraction methods, such as radiomics or deep learning‐based image analysis, to reduce operator dependence and enhance robustness.

Finally, the inclusion of additional biomarkers and patient‐specific variables—such as genomic, molecular, or treatment‐related factors—should be considered in future work to further improve predictive accuracy and clinical applicability. Further research will also evaluate the model's effectiveness across more diverse demographic and clinical subgroups.

## 6 Conclusion

Compared with ultrasound assessment alone, the random forest model combining ultrasound imaging and clinical features significantly improved the accuracy of pCR prediction and showed better predictive performance than conventional MRI assessment in this cohort. The integration of multidimensional data may assist clinical decision‐making after further prospective multi‐center external validation.

## Author Contributions

**Xiaohui Ji:** methodology, supervision, writing – review and editing. **Tongtong Hao:** methodology, software, data curation, validation, visualization, writing – original draft, writing – review and editing. **Mengying Wei:** writing – review and editing, validation. **Qianying Zhao:** data curation, validation.

## Funding

The authors have nothing to report.

## Ethics Statement

The study was conducted in accordance with the Declaration of Helsinki and its subsequent amendments. Ethical approval was obtained from the Ethics Committee of the Fourth Hospital of Hebei Medical University (Approval No. 2024KS207). Given the retrospective nature of the study, the requirement for individual informed consent was waived.

## Conflicts of Interest

The authors declare no conflicts of interest.

## Supporting information


**Table S1:** Key Regression Coefficients of the Logistic Regression Model.
**Table S2:** Comparison of Variable Distributions Between Development and Validation Cohorts.

## Data Availability

The data that support the findings of this study are not publicly available due to concerns regarding participant privacy and confidentiality. However, the data may be made available from the corresponding author upon reasonable request and with approval from the Ethics Committee of the Fourth Hospital of Hebei Medical University.
