# Supplementary material for: Ultrasound and Clinicopathological Features‐Based Machine Learning Model for Predicting Neoadjuvant Therapy Efficacy in Breast Cancer
Source: Cancer Rep (Hoboken). 2026 Jun 16;9(6):e70600. doi: 10.1002/cnr2.70600 (PMC13271844; doi:10.1002/cnr2.70600)
Supplement: Supplementary file 1 — Table S1: Key Regression Coefficients of the Logistic Regression Model. Table S2: Comparison of Variable Distributions Between Development and Validation Cohorts. [file CNR2-9-e70600-s001.docx]

**Supplementary Materials**

**(1) Model Development & Hyperparameter Tuning**

We developed and validated a prediction model using three machine learning algorithms: Random Forest, Logistic Regression, and Support Vector Machine (SVM). The model-building process included the following key steps:

**Data Preparation:**

The dataset used for model development contained multiple predictors, including categorical variables such as *Early_Vol_cat, echo, subtype*, and continuous variables such as *TILs.* The outcome variable was *pcr.*All categorical variables were encoded using Label Encoding.

**Model Selection:**

1. The logistic regression model predicts the probability of the positive class (PCR = 1) using the logistic function: $P\left( y=1 | X \right)=\frac{1}{1+e^{-(\beta_{0}+\beta_{1}\cdot X_{1}+\beta_{2}\cdot X_{2}+\beta_{3}\cdot X_{3}+\beta_{4}\cdot X_{4})}}$*.* Regularization parameter 𝐶 and penalty type (L1 or L2) were optimized using grid search.
2. The Random Forest model, based on an ensemble of decision trees, predicts the class label by majority voting. The number of trees (*n_estimators*), tree depth (*max_depth*), and minimum samples for split (*min_samples_split*) were tuned using grid search.
3. The SVM model uses a linear or radial basis function (RBF) kernel for classification, with the decision boundary given by: $\omega\cdot X+b=0$. Regularization parameter 𝐶 and *kernel* type (*linear* or RBF) were optimized through grid search.

**Internal Validation:**

We used cross-validation (5-fold CV) during the grid search process to ensure the stability and generalizability of the models. The dataset was split into training and validation sets (70% training, 30% testing) using the *train_test_split* function.

**Grid Search on Training Set**

The following prediction models were developed and their key components are presented:

1.Random Forest Model:

The optimal model was identified through grid search. The hyperparameters for the model were:

- - - *n_estimators* = 300
    - *max_depth* = 20
    - *min_samples_split* = 5

2.Support Vector Machine Model:

The best SVM model was selected with the following hyperparameters:

- - - C = 10
    - *kernel = rbf*

Due to the complex structure of Random Forest and SVM models, full model coefficients are not presented. However, the models are available upon request or implementation using the specified hyperparameters.

3.Logistic Regression Model:

The Logistic Regression model was selected with the following hyperparameters:

- - - C = 1
    - *penalty* = l2
    - *solver = liblinear*

The prediction model can be used to estimate the probability of PCR using the logistic regression formula: $P\left( y=1 | X \right)=\frac{1}{1+e^{-(\beta_{0}+\beta_{1}\cdot X_{1}+\beta_{2}\cdot X_{2}+\beta_{3}\cdot X_{3}+\beta_{4}\cdot X_{4})}}$.

**Supplementary Table 1. Key Regression Coefficients of the Logistic Regression Model**

| Variable | Coefficient (β) |
| --- | --- |
| (Intercept) | 4.317 |
| Early-NAT Reduction Rate of Volume | 2.545 |
| echo = no change | Ref |
| echo = higher | 1.850 |
| echo = lower | -2.593 |
| echo = disappearance | 0.736 |
| Subtype=Luminal | Ref |
| Subtype=HER2 | 0.833 |
| Subtype=Triple negative | 0.926 |
| TILs=low | Ref |
| TILs=Intermediate/high | 1.0102 |

**(2) Model Evaluation on Validation Set**

**Threshold Determination for pCR Prediction**

For each machine learning model, the optimal probability threshold for classifying patients as pCR or non-pCR was determined using the following procedure. First, the receiver operating characteristic (ROC) curve was generated based on the predicted probabilities on the validation set. For each possible threshold, the true positive rate (TPR, sensitivity) and false positive rate (FPR, 1-specificity) were calculated, and Youden’s J statistic (J = TPR - FPR) was computed. The threshold corresponding to the maximum Youden's J value was selected as the optimal cutoff.

Using this approach, the optimal probability thresholds were determined as follows: 0.8196 for the Random Forest model, 0.7691 for the Logistic Regression model, and 0.8072 for the Support Vector Machine (SVM) model.

**(3) Sample Distribution in the Training and Validation Sets**

To evaluate the comparability between the training and validation cohorts in terms of key feature variables and the primary outcome variable (pCR), chi-square tests were performed for each categorical variable, along with the calculation of Cramer's V to assess the strength of association. The results showed no statistically significant differences in the distribution of these variables between the two cohorts, with all Cramer's V values below 0.1, indicating small effect sizes and overall consistency in variable distributions. Detailed results are presented in Table 2.

**Supplementary** **Table 2. Comparison of Variable Distributions Between Development and Validation Cohorts**

| Variable | Training Set (n = 426) | Validation Set (n = 183) | *p*-value | Cramer's V |
| --- | --- | --- | --- | --- |
| Early-NAT Reduction Rate of Volume | | | 0.934 | 0.004 |
| 80% or Below | 62.44% | 62.84% |  |  |
| Above 80% | 37.56% | 37.16% |  |  |
| echo |  |  | 0.625 | 0.042 |
| No change | 57.28% | 57.38% |  |  |
| higher | 22.30% | 25.68% |  |  |
| lower | 12.44% | 11.48% |  |  |
| disappearance | 8.00% | 5.46% |  |  |
| subtype |  |  | 0.679 | 0.035 |
| HER-2 | 59.62% | 62.84% |  |  |
| ER+ | 21.83% | 19.13% |  |  |
| Triple-negative | 18.54% | 18.03% |  |  |
| TILs |  |  | 0.395 | 0.036 |
| Intermediate/high | 63.62% | 60.11% |  |  |
| low | 36.38% | 39.89% |  |  |
| pCR(Positive) | 80.99% | 79.24% | 0.592 | 0.021 |
